# Transformative Learning, Priming, and Simulation Timing: A Randomized Controlled Pilot Study Among Emergency Medicine Residents

**DOI:** 10.7759/cureus.47567

**Published:** 2023-10-24

**Authors:** Timothy Y Khowong, Nehal N Khamis

**Affiliations:** 1 Department of Emergency Medicine, NewYork-Presbyterian Queens, Queens, USA; 2 Department of Population Health, Hofstra University, Hempstead, USA; 3 Department of Advanced Studies in Education/Master of Education for Health Professions Program, Johns Hopkins University, Baltimore, USA

**Keywords:** medical resident education, simulation in medical education, didactic lecture, lvad emergency, transformative learning, priming

## Abstract

Background

Traditionally, learning is thought to occur best when prerequisite cognitive background information is delivered before simulation training. More recent studies have attempted to analyze the transformative nature of simulation by placing simulation before didactics. However, these studies were flawed as they provided background on the subject before the simulation itself. Our study aims to isolate the transformative effect of simulation and answer the question of whether lecture or simulation should come first.

Methodology

We designed a novel simulation session and accompanying lecture for 18 Emergency Medicine residents in all three years of training regarding a subject they were entirely unfamiliar with, the emergent management of a left ventricular assist device (LVAD). The residents were randomized into two groups. One group had the lecture (8/18) before their simulation, while the other group (10/18) had the simulation first and the lecture afterward, testing the motivational nature. Thereafter, both groups responded to a post-session survey with Likert-style and open-ended comment questions to assess the reaction to the session and a knowledge-based multiple-choice question test.

Results

Both groups did not score significantly differently on either the immediate post-test or a retention post-test that we administered four weeks later. Three of eight participants reported in open comments that they were much more comfortable with a lecture-first than a simulation-first format.

Conclusions

Despite controlling for some of the limitations of previous studies, our results including learners’ preferences do not support a transformation in the sequence of clinical skills learning. Until other larger studies prove the opposite, we recommend continuing with the lecture followed by the simulation sequence as per existing conceptual simulation learning frameworks.

## Introduction

Medical simulation has long been a feature of Emergency Medicine training programs. Simulation training within Emergency Medicine utilizes carefully constructed scenarios and objects to allow for focused practice of specific tasks without risking patient harm. Multiple studies within the academic Emergency Medicine literature have shown that simulation increases learner engagement and retention of learned knowledge and skills compared to a traditional, lecture-based curriculum [1]. Broadly, there are two main styles of simulation used in Emergency Medicine, namely, the resuscitation scenario and the hands-on practice session. The resuscitation scenario presents a patient with a specific medical problem(s) and the healthcare team must take an accurate history and perform a physical examination, order the correct tests to diagnose the condition, and deploy the correct interventions to treat it. This use of simulation focuses on the knowledge-based learning portion of the STATS framework for simulation outlined by Aggarwal et al. [2] and the “Knows” and “Know How” levels of Miller’s pyramid in addition to the psychomotor skills (history and physical examination) component. The hands-on practice session is usually applied to a certain procedure or set of procedures. It comprises a teaching portion where the correct steps of a procedure are outlined followed by the opportunity for learners to practice on a training model. It focuses on the task deconstruction and training in the laboratory environment portions of the STATS framework and the “Shows” as well as “Knows How” levels of Miller’s pyramid.

Some specialties outside of Emergency Medicine have attempted to alter the sequence in the STATS framework by placing the knowledge-based learning after training in the laboratory environment, with variable reported levels of success that differed according to the methodology used. We believe that addressing some of their study design elements with modifications to isolate simulation and connect them to the frameworks of Miller’s pyramid, the STATS framework for simulation, transformative learning theory, and cognitive learning theory might yield more solid evidence for the correct sequence.

Transformative learning theory is the idea that there is a “disorienting dilemma,” or a situation that leads to a “spark” for learning. This may occur naturally when a person is faced with a challenging situation with high stakes and is forced to learn a new skill or concept to cope with or overcome this situation. Therefore, it follows that there may be a way for an educator to introduce a controlled version of the “disorienting dilemma” to learners and use it as a pedagogical tool. A recent review by Vipler et al. [3] analyzed the prevalence of transformative learning theory within the Graduate Medical Education literature and found that it is mostly used by primary care fields to teach concepts regarding professionalism or professional identity formation. Another study by Willis et al. [4] utilized a design that could be seen as transformative in nature. They allowed 42 students to either view an instructional video on laparoscopic suturing first (“instruction first”) or try to perform the task without instruction first (“struggle first”). The “struggle first” group was then allowed to view the instructional video afterward. Both groups were then compared to assess their ability to perform the taught task of laparoscopic suturing. The “struggle first” group scored significantly higher on their final test of suturing than the “instruction first” group. Additionally, older cognitive literature seems to support that there is value in working through a problem to arrive at a solution rather than just memorizing the correct answer and that unsuccessful attempts at solving a problem improve later learning [5,6]. Thus, transformative learning theory may have a place in Emergency Medicine, using simulation as the transformative experience to open the minds of learners to the information to follow.

Alternatively, priming is a well-known learning phenomenon and is nestled within cognitive learning theory. It is described as the phenomenon where exposure to a stimulus influences a response to a later stimulus, decreasing latency for recall and bringing up related concepts to the forefront of working memory to increase learning for the future [7]. A study by Wexler et al. [8] analyzed the performance of children on computerized math or reading content after playing short video games that were designed to prime executive functions, including pattern recognition and working memory. They found that the children who played the games before engaging with the computerized content performed significantly better than their peers, suggesting that priming can be a powerful tool to increase learning if the priming tool is carefully designed to activate the parts of the brain important for the next task. Another factor that may play into the power of priming is decreasing the cognitive load associated with learning. By repeating exposure to similar information using different modalities, the cognitive load of that information can be spread out and a person may have an increased ability to retain more information [9,10]. Additionally, Aggarwal et al., as mentioned before, have outlined the STATS framework of simulation which places “knowledge-based learning” as the prerequisite for engaging with psychomotor domain objectives in the simulation lab which is congruent with cognitive learning theory.

Existing research gives credence to both arms of this study design as valid improvements to either simulation or lecture alone. When compared head-to-head, there may be a signal toward transformative learning as a more powerful tool for education. Thus, our study aims to test the hypothesis that attending simulation first may play an important role as a transformational primer tool for asserting the later learning of relevant knowledge. The research question that our study tried to answer is whether starting with simulation training before the cognitive background lecture would result in better gain and retention of knowledge among Emergency Medicine residents.

## Materials and methods

This study aimed to pit transformative learning theory and priming within cognitive learning theory against each other. The transformative group experienced a simulation with a topic they were unfamiliar with, followed by a full didactic lecture on managing that type of patient. The priming group had the didactic first, followed by the simulation. It was imperative that the subject matter of the simulation and lecture was a topic that the participants were unfamiliar with to prevent a spoiler effect of prior knowledge on the transformative nature of the simulation. Therefore, the subject of management of a patient with a left ventricular assist device (LVAD) was chosen for this study as our hospital is not an LVAD center and patients with LVADs rarely come to our hospital rather than going to the LVAD-capable centers that are in our region. The study design was reviewed and approved by the NewYork-Presbyterian Queens Institutional Review Board (approval number: 14390422).

Participants

A total of 18 subjects were recruited on the day of the study, randomized based on PGY year of training, and divided equally into either the lecture-first or simulation-first groups. In total, 12 males, five females, and one non-binary person participated. Participant ages ranged from 26 to 33 years. Racial and ethnic origin were primarily Asian-American, Indian, Hispanic, and Caucasian, which is representative of the current ethnic breakdown of our program. Only current resident physicians at our institution were included in this pilot study. Overall, seven PGY-1 residents, three PGY-2 residents, and eight PGY-3 residents participated in the study. Notably, the vast majority of the residents reported having never/rarely taken care of a patient with an LVAD or received training on them before.

Measures

We used two data collection instruments; the first instrument consisted of a seven-question post-session evaluation survey utilizing five-point Likert-scale questions to evaluate the sessions in terms of perceived utility of the session and skills learned, realism of the case, and confidence in performing skills gained. A free text box for open-ended feedback was included as well.

The second data collection instrument we utilized included two multiple-choice question (MCQ) tests, both of which included 20 questions. Unfortunately, validated question sets specifically geared toward the evaluation and management of LVADs were non-existent at the time of the creation of the study, so these questions were generated by the first author (TK) and three other Emergency Medicine-trained physicians with experience with LVAD patients. They were written in the format of board exam questions and were validated by four other clinical faculty members for content and discriminative evidence of construct validity. The survey and MCQ tests are attached in Appendix A.

Procedure

Emergency Medicine residents were recruited on the day of the study during their weekly didactic conference. They consented to participate in the study in person with both a paper and electronic form available. After consent was obtained, participants were first sorted by postgraduate year of training, then randomized simply into two groups using a random number generator. This left us with a representation of each class per group. One group participated in the simulation first, and the other had the didactic lecture first. This study was unblinded due to the nature of the variable being studied.

Briefly, the case scenario is of a patient with an LVAD who has a driveline infection leading to septic shock, which leads to a suction event of the device, and, ultimately, cardiac arrest. Learners must appropriately evaluate the device (a high-fidelity simulator attached to the simulation mannequin), obtain vital signs, and verbalize/act out their resuscitation of the patient. No instruction was provided by the faculty during the simulation itself and the debriefing after the simulated resuscitation was limited to the discussion of team dynamics and interpersonal communication skills.

On the other hand, the lecture provided information on the components of the device, how to evaluate a patient with one of these devices, common complications, and management of those complications (Knows and Knows How levels of Miller’s pyramid). The full lecture and simulation write-up are included as Appendices A and C. After participation in either the lecture or simulation session, the residents then participated in the activity they had not yet attended. Afterward, all participants were instructed to fill out the post-session survey in an electronic format utilizing Google Forms. It was expected that they would be able to complete the post-session survey within 30 minutes. Four weeks later, a second MCQ test (Appendix B) using similar questions as the first was distributed to the participants to assess their retention.

Statistical analysis

Grading for the MCQ tests was done as a simple total percentage of correct answers with all questions having the same weight. The percentages were averaged among the group and compared with a t-test, comparing the groups that had simulation first or lecture first for both the immediate post-test and four-week retention test. Likert scale results were collected and expressed as a simple mean and standard deviation. Open-ended responses were collated and coded before being analyzed for common themes.

## Results

A total of 18 residents participated in the session (simulation and lecture, regardless of the sequence). Their characteristics are listed in Table 1. Eighteen participants completed the initial post-session survey and MCQ test, while 14 participants completed the four-week retention MCQ test, with an attrition rate of 22.2%. Within the post-session survey, eight participants had the lecture first, while 10 had the simulation first. Of those who responded to the retention survey, six had the lecture first, while eight had the simulation first.

**Table 1:** Summary of participant characteristics. LVAD = left ventricular assist device

Total number of participants	18
Lecture first	8
Simulation first	10
Male	12
Female	5
Non-binary/Third gender	1
PGY-1	7
PGY-2	3
PGY-3	8
Have had little prior training on the management of LVADs	15
Have had some prior training on the management of LVADs	3
Have had a lot of prior training on the management of LVADs	0
Have rarely/never managed a patient with an LVAD	18
Have managed some patients with an LVAD	0
Have managed many patients with an LVAD	0

**Figure 1:**
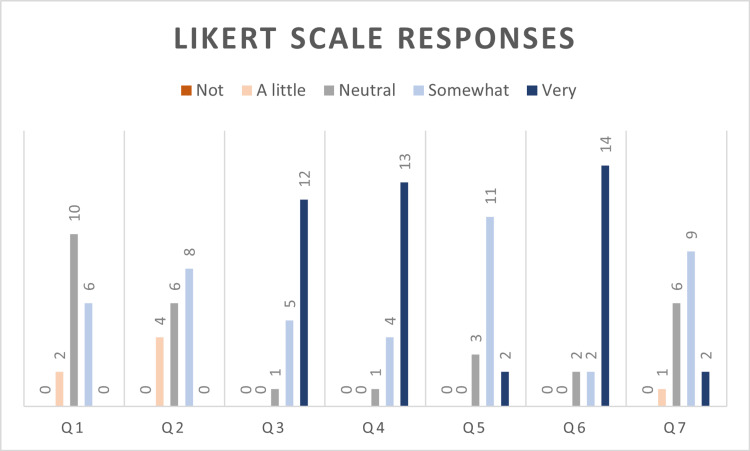
Summary of Likert-scale data from the post-session survey.

All participants’ responses to the post-session evaluation survey were positive. The results are summarized in Table 2, while Figure 1 visualizes the breakdown of their responses. They felt at least somewhat confident in their ability to evaluate, treat, and apply interventions to treat a patient with an LVAD, with all mean scores above 3. They rated their level of confidence the highest regarding their ability to provide effective transitions of care/hand-offs when caring for LVAD patients and when evaluating a patient with LVAD (mean = 4.67 ± 0.67 and 4.61 ± 0.59, respectively). They also perceived the role of the didactic and simulation sessions (prompts 6, 7, and 10-12 in Appendix A) as helpful in increasing their knowledge about the topic with a high mean of agreement of 4.67 ± 0.58 that the didactic lecture provided a mental framework to evaluate patients with LVADs.

**Table 2:** Summary of results of the attitude portion of the survey. LVAD = left ventricular assist device

	Prompt	Mean	SD
Q1	After participating in the session, I feel confident in evaluating all of the components of an LVAD	3.22	0.63
Q2	After participating in the session, I feel confident obtaining blood pressure in a patient with an LVAD	3.22	0.79
Q3	After participating in the session, I feel confident evaluating a patient with an LVAD	4.61	0.59
Q4	The didactic provided a mental framework for me to evaluate patients with LVADs	4.67	0.58
Q5	The didactic helped answer questions I had about LVADs	3.83	0.6
Q6	When caring for LVAD patients, I am confident in my ability to provide effective transitions of care/hand-offs	4.67	0.67
Q7	I feel confident in applying immediate interventions to manage a critically ill LVAD patient	3.67	0.75

The data for the MCQ exams is listed in Table 3 and Table 4. On the MCQ exams, the lecture-first group scored at a mean of 82.5% (SD = 12.2) on the post-session exam and 74.2% (SD = 15.3) on the retention exam. The simulation group scored at a mean of 86.5% (SD = 5.3) on the post-session exam and 74.4% (SD = 9.8) on the retention exam. The mean difference between the groups on the post-session exam was -4, with a t of 0.935 (p = 0.182). The mean difference between the groups on the retention exam was -0.2, with a t of 0.031 (p = 0.5). The difference between groups was not statistically significant either immediately post-session or at four weeks.

**Table 3:** Summary of results for multiple-choice questions exam portion of the survey.

	N - immediate	Mean score (%) - immediate	SD	N - retention	Mean score % - retention	SD
Lecture first	8	82.5	12.2	6	74.2	15.3
Simulation first	10	86.5	5.3	8	74.4	9.8

**Table 4:** Independent-sample t-test of exam score difference between lecture- and simulation-first groups. *: p < 0.05 indicates statistical significance.

	Mean difference (%)	t	df	P-value*	95% CI
Immediate	-4	0.935	16	0.182	(-13.1–5.1)
Retention	-0.2	0.031	12	0.5	(-14.8–14.4)

Eight participants provided written, qualitative feedback. Three stated that they would have preferred to have the lecture before the simulation, while none commented that they would have preferred the simulation first. The remainder stated that they would have preferred smaller groups for the simulation sessions.

## Discussion

The results of the current study show that the sequence of a paired simulation and lecture did not change the level of knowledge learned and retained by participants immediately and after four weeks.

Based on prior study results investigating the best sequence of the lecture and simulation training [4,11-13], we were interested in investigating if participants would still learn and retain knowledge better when they participate in simulation first if no prerequisite knowledge is mentioned in the simulation session.

Examining the methodology of the relevant published studies with results in favor of the simulation-first model, we found that McCoy et al. studied simulation vs. lecture in a crossover design fashion and found simulation to be superior to lecture; however, they did not study them in combination, so it is difficult to extrapolate their findings to ours [11]. The Zendejas group used a similar design with surgical residents in all three years of their training over nine different sessions before combining their data [13]. They found that learners who had the simulation first had statistically significant improvements in their knowledge, as measured by a multiple-choice questionnaire. They justified their result by mentioning that “Participating in a simulated scenario before receiving didactic information could activate prior knowledge and set a foundation for new knowledge.” The Thampi group used a similar design once again, but only to teach the psychomotor skill of transesophageal echocardiography, rather than cognitive learning objectives [12]. They found that immediate post-test scores were the same, but the retention in the simulation-first group was significantly improved compared to the lecture-first group. Thampi et al. built their argument on their belief that “theoretical framework behind the activation of prior knowledge through engaging the learner in a ‘mind primer’ (simulated scenario) has a stronger foundation rooted in theories of cognitive psychology.” So, the theory of activation of prior knowledge [14] was central to the assumptions and justifications of the results of these studies. In our methodology, we were keen not to provide any foundational relevant knowledge by the facilitators in the simulation sessions so that the simulation-first group was not subjected to knowledge before the didactic lecture. If there is knowledge provided during the simulation-first session, followed by activation of the knowledge during the following lecture, then this would represent subjecting participants to knowledge during the simulation (a mix of knowledge and skills). This would mean testing the integration of didactic with simulation rather than looking at the transformative role of simulation.

Stefaniak and Turkelson [15] conducted their study with critical care nurses in a paired lecture and simulation regarding advanced hemodynamic monitoring. Importantly though, this was included as part of an entire week-long course which included other prior lectures and pre-reading. They found no statistically significant difference in MCQ exam scores between their two groups, and their groups expressed a preference for having didactic before simulation; results that are concordant with ours despite the difference in methodology.

An important factor to consider when interpreting our findings is the feedback from the participants stating that the large group simulation felt disjointed and crowded. This is understandable given that the usual simulation environment has four to five participants while this one had ten participants, double what they are used to. It is possible that the increase in the number of participants decreased the transformative experience as it became more chaotic and diffused the responsibility of leading the simulated resuscitation. Repeated versions of this experiment should consider reducing the group size to five participants to preserve the small-group learning benefits of simulation.

**Figure 2:**
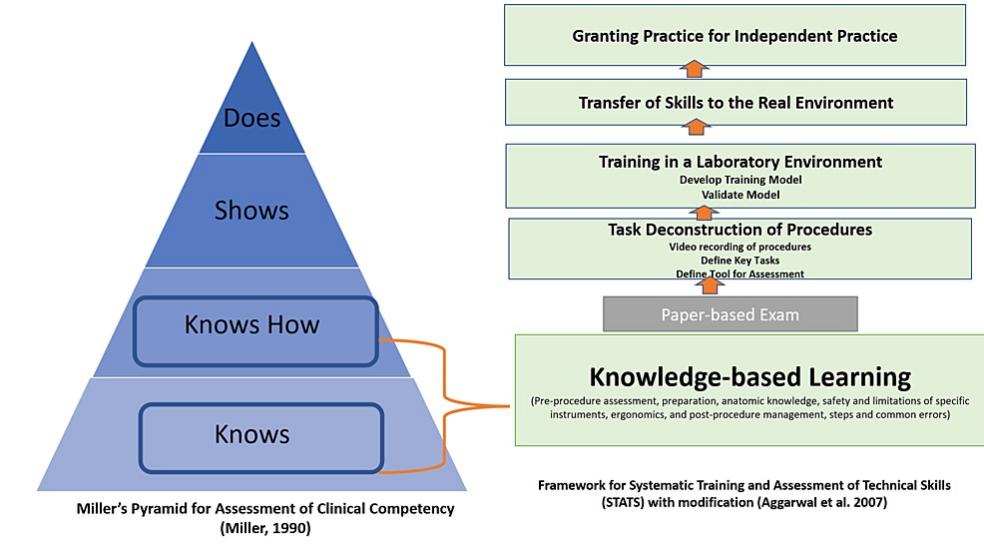
Correlation between Miller’s pyramid and the STATS framework for simulation. Adapted from Miller et al. [16] and Aggarwal et al. [2] and created by N. Khamis.

Although our results were equivocal regarding the sequence, our participants’ feedback and perception of the didactic, as providing a mental framework to evaluate patients with LVADs, are consistent with the conceptual frameworks for developing, teaching, and assessing clinical skills and procedures, e.g., Miller’s pyramid [16], Fitts and Posner’s three-stage theory of motor skill acquisition [17] (cognition, association, and automation), and STATS. The STATS framework by Aggarwal et al. outlines the theoretical underpinnings of the use of simulation in education and displays an idealized framework for it. The correlation between Miller’s pyramid and the STATS framework is visualized in Figure 2. These frameworks emphasize the importance of learning prerequisite knowledge (at the Knows and Knows How levels of Miller’s pyramid), followed by passing a knowledge test before starting the simulation training.

However, as Aggarwal et al. stated “the reality of how one learns is far removed from the simplistic notion of acquiring procedure-specific knowledge. Learning and educational theories have been proposed in an attempt to make students learn more effectively.” Relevant theories include the theories of activation of prior knowledge [18], Kolb’s Cycle of experiential learning [19], and the transformative learning theory, which we tried to investigate while controlling for knowledge delivery by the instructors. In addition, all of our participants reported that they have rarely/never managed patients with LVADs.

In addition to the crowded simulation sessions reported by our respondents, this study is limited by the small number of only 18 participants. Future improvements could be made by repeating it with a larger sample size, at other academic sites, or within the same system with different cases and topics to improve the power of the study. Improving the power might be able to show a difference between the two groups. It could also help provide more evidence regarding learners’ preferences. Three out of the eight participants in our study who provided qualitative feedback stated that they preferred the lecture-first approach. If the learning is equivocal between the two groups, then learner preference for comfort should be considered when designing paired lecture and simulation sessions and the lecture should probably be given first.

So, the question still remains: should we change the current best practice of the prerequisite knowledge lecture preceding simulation training, or should we be encouraged to utilize the transformational role that simulation can offer and start with simulation first?

## Conclusions

We have attempted to utilize simulation as a transformational learning tool rather than as a teaching tool to see if it would inspire learners to become more invested in traditional cognitive learning. Despite controlling for some limitations of the previous studies, including the spoiler effect of providing prior knowledge, our results, including learners’ preferences, do not support a deviation from the current sequence of clinical skills that places cognitive learning before simulation-based learning. Our study was limited by a small sample size but showed no difference between the two groups both immediately after and in the retention exam, supporting the existing simulation framework. Additionally, learners expressed feelings of discomfort with the transformational strategy, perhaps showing an undesirable side effect of transformational learning which runs counter to the psychological safety usually required for learning. Until other larger studies prove the opposite, we recommend continuing with the lecture followed by the simulation sequence as per existing conceptual simulation learning frameworks. This is to continue until strong supportive evidence for the transformative simulation-first model evolves from larger studies with appropriate non-confounded methodologies.

## Appendices

Appendix A. Post-session survey and exam

**Table 5:** Post-session Likert-scale questions and knowledge exam.

LVAD session post-survey
Please answer the following prompts to the best of your ability
1. Did you have the lecture first or the simulation first?
a. Lecture first
b. Simulation first
2. Please indicate your level of training
a. PGY-1
b. PGY-2
c. PGY-3
3. Please indicate your gender identity
a. Male
b. Female
c. Third gender/non-binary
d. Prefer not to say
e. Other
4. Please choose the option below that fits you the best
a. I have had little prior training on the management of LVADs
b. I have had some prior training on the management of LVADs
c. I have had a lot of prior training on the management of LVADs
5. Please choose the option below that fits you the best
a. I have rarely/never managed a patient with an LVAD
b. I have managed some patients with an LVAD
c. I have managed many patients with an LVAD
Attitudes
6. Following this course, I feel more confident in applying immediate interventions to manage a critically ill LVAD patient
Strongly agree
Agree
Neutral
Disagree
Strongly disagree
7. Following this course: I feel more confident in my ability to provide effective transitions of care/hand-offs
Strongly agree
Agree
Neutral
Disagree
Strongly disagree
8. The didactic helped answer questions I had about LVADs
Strongly agree
Agree
Neutral
Disagree
Strongly disagree
9. The didactic provided a mental framework for me to evaluate patients with LVADs
Strongly agree
Agree
Neutral
Disagree
Strongly disagree
10. After participating in the session, I feel confident evaluating a patient with an LVAD
Strongly agree
Agree
Neutral
Disagree
Strongly disagree
11. After participating in the session, I feel confident obtaining blood pressure in a patient with an LVAD
Strongly agree
Agree
Neutral
Disagree
Strongly disagree
12. After participating in the session, I feel confident in evaluating all of the components of an LVAD
Strongly agree
Agree
Neutral
Disagree
Strongly disagree
13. Can you list any changes you will make to your practice based on this session?
14. How could this simulation and didactic session be improved?
Knowledge Questions
15. A 62-year-old man with an LVAD presents to the emergency department with worsening fatigue. On physical exam, you note patient appears pale. Later, the lab calls you to notify you that the hemoglobin is 5.2 and there are schistocytes on the peripheral blood smear. The patient’s stool occult is negative. What lab test will help you diagnose the cause of this patient’s anemia?
a. Folate
b. LDH
c. B12
d. Transferrin
16. A 65-year-old woman with a left ventricular assist device presents to the emergency department with altered mental status via ambulance. She does not respond to verbal or painful stimuli. What is the first step in evaluating this patient?
a. Auscultate the precordium
b. Obtain blood pressure with a Doppler signal
c. Obtain an ECG
d. Palpate for a pulse
17. How do you obtain accurate blood pressure in patients without a pulsatile blood pressure
a. Impossible
b. Manually inflate the cuff around the upper arm and listen with a stethoscope at the brachial artery
c. Manually inflate the cuff around the upper arm and apply US Doppler to the radial artery
d. The blood pressure is calculated by looking at the setting on the LVAD external system controller
18. What is the indication for LVAD placement?
a. A bridge to cardiac transplantation
b. A bridge to recovery (reversible pathology)
c. Destination therapy or long-term treatment of patients who are not candidates for transplant
d. All of the above
e. Options A and B only
19. A 54-year-old female with a history of CHF and LVAD placement one year ago presents with left arm weakness and facial droop. Which of the following is the most likely cause of the patient’s presentation?
a. Upper extremity DVT
b. SAH
c. Ischemic stroke
d. B12 deficiency
20. What does the PI (Pulsatile Index) seen on the LVAD settings refer to?
a. Estimation of cardiac output
b. It is related to the amount of contractility of the native heart, as well as preload entering LVAD
c. The speed at which the LVAD rotors are spinning
d. The amount of wattage to maintain the normal speed and flow
21. Which of the following matches the LVAD component with the correct function?
a. Pump - The battery that powers LVAD
b. Driveline - Connection to the external controller
c. Inflow cannula - Pushes blood into the aorta
d. System Controller - Generates the blood flow
22. A 60-year-old man with a left ventricular assist device presents to the Emergency Department by EMS because his LVAD is alarming. He has been having multiple episodes of vomiting and diarrhea for the past few days. His MAP is 55 mmHg by Doppler and his mucous membranes appear dry on exam. What is the most likely cause of his alarm?
a. Pump thrombus
b. Sepsis
c. Suction event
d. Low battery
23. True or false: It is safe to cardiovert/defibrillate with an LVAD in place
a. True
b. False
24. You are managing a patient with a left ventricular assist device and note that they have hypotension. The device appears to be functioning properly. Which of the following is the next best step in management?
a. Amiodarone
b. Normal saline IV bolus
c. Norepinephrine
d. Dobutamine
25. A 70-year-old woman with a recently placed left ventricular assist device presents because she has been having fevers. Which of the following physical exam findings is most concerning?
a. Normal exam
b. Suprapubic tenderness
c. Focal rhonchi
d. Erythema and purulence at the drive line site
26. A 26 female with a history of peripartum cardiomyopathy with LVAD placed 6 months ago as a bridge to transplant presents for SOB for 3 days. Her MAP by Doppler is measured at 55 mmHg. She has not been taking her prescribed medications due to recent issues with insurance. Her blood work is significant for hemoglobin of 9.4, INR of 1.2, PT of 12, and aPTT of 31. Which of the following changes in pump settings would you expect to see?
a. Increased flow
b. Decreased power consumption
c. Increased power consumption
d. No change
27. Patients with an LVAD and in cardiac arrest require unmodified high-quality CPR, despite potential damage to the device
a. True
b. False
28. The most common LVAD complication is a suction event
a. True
b. False
29. A 37 male with a history of post-viral myocarditis with LVAD placement is BIBEMS in cardiac arrest. The patient is placed on pads and monitored, intubated for airway protection, and access is obtained. You do not auscultate a precordial hum on the cardiac exam. What is the most likely cause of the patient’s presentation?
a. Hemorrhagic stroke
b. Pulmonary embolism
c. Device battery failure
d. Myocardial infarct
30. A 61 male with a history of ischemic heart disease and LVAD placement arrives in ED for generalized weakness for one day. He denies chest pain, abdominal pain, or fevers. His vitals are MAP of 60 mmHG by Doppler, HR of 145, RR of 14, 94% O_2_ on room air, and 36.8°C. The patient appears alert and calm. LVAD is not alarming and settings are normal. ECG demonstrates ventricular tachycardia. What is the appropriate treatment?
a. Amiodarone IV
b. Synchronized cardioversion
c. Defibrillation
d. Magnesium IV
31. A 63 male with a history of non-ischemic dilated cardiomyopathy LVAD placed one year ago as a bridge to transplant presents for altered mental status. The patient is awake and alert; however, he is unable to participate in history taking due to his mental status. He makes repeated attempts to climb out of the stretcher and requires chemical sedation. According to the daughter at the bedside, the patient was completely normal this morning when she spoke to him on the phone. Vitals show MAP by Doppler of 70 mmHG, HR 50s, and 92% pulse oximetry on room air. You auscultate LVAD hum over precordium. Which test will help you determine the etiology of the patient’s presentation?
a. CXR
b. CTA Chest
c. Echo
d. CT head
32. A 32 female with a history of mitral valve regurgitation and heart failure presents with pain, weakness, and fever following LVAD implantation. Vital signs show MAP by Doppler of 65 mmHG and temperature of 39.1°C. What is her treatment?
a. Oral cephalexin and discharge
b. IV Ceftriaxione and metronidazole and admission
c. IV Ceftriaxone and vancomycin and admission
d. IV Cefazolin and vancomycin and transfer to LVAD center
33. A 43 female with a history of SLE and restrictive cardiomyopathy with LVAD is BIBEMS after a syncopal event. The patient states that she collapsed this morning while getting ready for work. She is compliant with all of her medications. She still has her periods and is currently having heavy, prolonged menstruation. On exam, you see a tired-appearing female with pale conjunctiva. What is the likely etiology of her syncope?
a. Sepsis
b. Intracranial hemorrhage
c. Arrhythmia
d. Blood loss anemia
34. A 68 male with LVAD is brought into the ED in cardiac arrest. After the patient is placed on a monitor and pads you note at the pulse check that he is in PEA arrest. You auscultate a precordial hum. The LVAD is alarming. You note the pump power is 13 watts and flow is 2. Bedside US demonstrates dilated LV. What is the next step in management?
a. Fluid resuscitation
b. tPA
c. Turn the device off and on
d. Pericardiocentesis

Appendix B. Four-week retention exam

**Table 6:** Knowledge Retention Exam

LVAD session knowledge retention
Please answer the following prompts to the best of your ability
1. What year of training were you in during the initial session?
a. PGY-1
b. PGY-2
c. PGY-3
2. Did you participate in the lecture first or the simulation first?
a. Lecture first
b. Simulation first
3. A 73-year-old man presents to the emergency department with AMS. He Does not respond to verbal or painful stimuli. What is the first step in evaluating this patient?
a. Check for a pulse
b. Auscultate the chest
c. Obtain a CXR
d. Obtain blood pressure with a Doppler
4. Which of the following patients is indicated to receive LVAD placement?
a. A patient with cor pulmonale from severe COPD
b. A patient who is a candidate for cardiac transplantation
c. A patient diagnosed with reversible myocarditis
d. All of the above
e. B and C only
5. What does the PI (Pulsatile Index) seen on the LVAD setting refer to?
a. The power to maintain normal speed and flow
b. Cardiac output
c. The contractility and preload of native heart
d. The speed at which the LVAD rotors are spinning
6. You are managing a patient with a left ventricular assist device and note that they have hypotension. The device appears to be functioning properly. What is the next best step in management?
a. The battery is low
b. Suction event
c. Pump thrombus
d. The power is low
7. A 33-year-old female with a history of Takotsubo with LVAD placed 7 months ago presents for SOB for 3 days. Her MAP is measured at 50 mmHg. Her bloodwork is significant for hemoglobin of 8.9, INR of 1.2, PT of 12, and aPTT of 31. Which of the following changes in pump settings would you expect to see?
a. Increased flow
b. Decreased power consumption
c. Increased power consumption
d. No change
8. True or false: The most common LVAD complication is bleeding.
a. True
b. False
9. A 67 male with a history of ischemic heart disease and LVAD placement comes to ED for generalized weakness. He is awake and in no acute distress. His vitals are MAP 55 mmHG by doppler, HR 140, RR 14, 95% SaO2 on rm air and 36.8 C. LVAD is not alarming and settings are normal. ECG demonstrates the ventricular tachycardia. What is the appropriate treatment?
a. Synchronized cardioversion
b. Defibrillation
c. Amiodarone IV
d. Procainamide IV
10. A 43 female with a history of peripartum cardiomyopathy with LVAD implantation 2 weeks ago presents with a fever to your small community hospital. You inspect the drive line site and observe purulent discharge. What is her management?
a. Oral antibiotics and follow-up with her CT surgeon in 24 hours
b. IV antibiotics and admission
c. IV antibiotics and stat infectious disease consultation
d. IV antibiotics and transfer to LVAD center
11. A 63-year-old male with LVAD is brought into the ED in cardiac arrest. The patient is placed on the monitor and pads and you note at the pulse check that he is in PEA arrest. The LVAD is alarming and you note that the pump is 16 watts and the flow is 1.5. US demonstrates dilated LV. What is the next step in management?
a. tPA
b. Defibrillation
c. Fluid resuscitation
d. Call the in-house CT surgeon
12. A 58-year-old man with an LVAD presents to the emergency department with worsening fatigue. On exam, you note skin pallor. Labs are significant for hemoglobin of 4.9. The peripheral blood smear notes the presence of schistocytes. His stool occult is negative. What lab test will help you diagnose the cause of this patient's anemia?
a. Transferrin
b. Haptoglobin
c. Lactate
d. Folate
13. How do you obtain accurate blood pressure in patients without a pulsatile blood pressure?
a. The blood pressure is shown on the LVAD external system controller
b. Manually inflate the cuff around the upper arm and listen with the stethoscope at the brachial artery
c. Manually inflate the cuff around the upper arm and apply US Doppler to the radial artery
d. The only way is with an arterial line
14. A 65-year-old female with a history of CHF and LVAD placement two years ago presents with right arm weakness and facial droop. Which of the following is the most likely cause of the patient’s presentation?
a. Intraparenchymal bleed
b. Ischemic stroke
c. Upper extremity DVT
d. Seizure
15. True or false: It is safe to cardiovert/defibrillate with an LVAD in place?
a. True
b. False
16. A 68-year-old woman with a recently placed LVAD presents with fevers. Which of the following physical exam findings is most concerning?
a. Suprapubic tenderness
b. Focal rhonchi
c. Erythema and purulence at the drive line site
d. Normal exam
17. Patients with an LVAD and in cardiac arrest require unmodified high-quality CPR
a. True
b. False
18. A 42-year-old male with a history of post-viral myocarditis with LVAD placement is BIBEMS in cardiac arrest. You do not auscultate a precordial hum on the cardiac exam. What is the most likely cause of the patient’s presentation?
a. Myocardial infarct
b. Device battery failure
c. Pump thrombus
d. Hemorrhagic stroke
19. An 82-year-old male with a history of non-ischemic dilated cardiomyopathy with LVAD placed one year ago presents for altered mental status. His eyes open to noxious stimuli and he is making groaning sounds, however moves all extremities spontaneously. He was reportedly grocery shopping this morning when he suddenly fell to the ground. Vitals show MAP of 70mmHG, HR 40s, and 92% pulse oximetry on room air. Which test will help you determine the etiology of the patient’s presentation?
a. CXR
b. CTA Chest
c. Echo
d. CT head
20. A 47-year-old female with a history of restrictive cardiomyopathy with LVAD is BIBEMS after a syncopal event. The patient states that she collapsed this morning while getting ready for work. She still has her periods and is currently having heavy prolonged menstruation. On exam, you see a tired-appearing female with pale conjunctiva. What is the likely etiology of her syncope?
a. Sepsis
b. Arrhythmia
c. Intracranial hemorrhage
d. Blood loss anemia

Appendix C. Simulation case

**Table 7:** Simulation case information.

Simulation case title: Suction event in a patient with an LVAD: a simulation case for Emergency Medicine residents. Authors: Rozalyn Hesse, MD; Timothy Khowong, MD. Learner audience: Emergency Medicine Residents	
Patient name: Vladimir Hart. Patient age: 59. Chief complaint: Syncope, weakness. Physical setting: Simulation Lab	
Brief narrative description of the case	A 59-year-old male presents after a syncopal episode today in the setting of dehydration from gastrointestinal illness. On initial assessment, the team will need to work out how to evaluate an LVAD patient, including obtaining vital signs and blood pressure with Doppler. They will be practicing on a high-fidelity simulation mannequin which will be augmented with a proprietary LVAD simulator that utilizes a microcontroller. They will discover a low mean arterial pressure and a low-grade fever. IV fluids should be given; however, in this case, access is difficult and fluids are delayed. After the history, physical, and workup are initiated, the LVAD will experience a suction event and the appropriate alarms will go off. The patient will progress to a PEA arrest requiring resuscitation with ACLS, including intubation. ROSC will be achieved when sufficient fluids are administered
Primary learning objectives	By the end of this activity, learners will be able to assess a patient with LVAD in relation to their hemodynamic status and device function. Manage a patient who is having a device-related emergency, specifically a suction event. Provide handoff for a patient with an LVAD who has undergone emergency stabilization
Critical actions	Confirm LVAD is functioning by auscultation of the chest wall for pump sound. Obtain blood pressure with a manual cuff and Doppler. Inspect the driveline site in a sterile fashion. Inspect the controller of the device for device status and alarms. VAD coordinator contact was attempted and reattempted until completed. Recognize suction event and give IV fluids. Give appropriate chest compressions during cardiac arrest. Intubate. Coordinate transfer to the VAD center
Learner preparation or prework	This simulation case is part of a larger study examining the potential impact of the sequence of a paired simulation and didactic. Participants may have had no preparation or may have had an entire didactic just before the case regarding the evaluation and management of LVADs

**Table 8:** Initial patient presentation during the case.

Initial presentation			
Initial vital signs	HR: 95, BP: Undetectable, RR 20, SpO_2_ 95% RA, T 37.9		
Overall setting and appearance	Learners are in the simulation lab. The mannequin is dressed in a gown and the high-fidelity LVAD simulator is attached to its torso and left arm and is visible. He is moaning, but there is no other moulage on the mannequin. The patient will be moaning, lethargic, and slow to respond		
Standardized participants (and their roles in the room at case start)	At least 5 participants will be present in the case Team leader – Delegates roles and directs decision-making. Airway – evaluates patency and adequacy of the airway and provides interventions if necessary. Right-sided procedures and history + physical exam taking – stands on the right side of the patient, performs IV access and any other right-sided procedures that must be done. Will also obtain history and physical exam of patient Left sided procedures and evaluation of device – stands on the left side of the patient, examines the LVAD, performs any left-sided procedures that must be performed Floater for medications and extra tasks – no specific location, can call consults, grab medication, be responsible for Zoll when the patient has a cardiac arrest		
HPI	Volunteered: A 59-year-old male with a history of CAD/CHF with LVAD placed 2 years ago. He had an episode of syncope this morning after sitting up from bed. Woke up rapidly after laying back down in bed. No palpitations or chest pain. Must ask: Past two days, he has had 2 days of GI symptoms including multiple episodes of NBNB vomiting and watery diarrhea. Subjective fever and chills. He did not hit his head with the syncope this morning. The device has felt warm today but has not alarmed.		
Past medical/Surgical history	Medications	Allergies	Family History
CAD, CHF, LVAD, pacemaker	Atorvastatin, aspirin, warfarin, furosemide, metoprolol, amlodipine	NKDA	Noncontributory
Physical Examination			
General	Lethargic, muffled groaning		
HEENT	Atraumatic head, oropharynx clear		
Neck	Normal		
Lungs	Normal		
Cardiovascular	Mechanical hum. Tachycardiac. No palpable pulses		
Abdomen	Soft, non-distended. The driveline site appears clean, dry, and intact		
Neurological	No facial droop. Open eyes to pain, groaning, and withdraws to pain		
Skin	Cool, dry		
GU	Normal		
Psychiatric	Unable to evaluate		

**Table 9:** Instructor notes - changes and case branch points.

Intervention/Time point	Change in case	Additional information
3 minutes into the case	The patient will become even less responsive if no attempt to evaluate the device has been made	RN alerts the provider: “Doctor, the patient doesn’t look good. I think there’s something on his chest”
5 minutes into the case		The wife should prompt the team to call the LVAD coordinator, if not done so
10 minutes into the case	The patient becomes totally unresponsive; NSR is on the monitor but not Dopplerable BP. LVAD controller should display low flow and high power	RN should alert the provider: “Doctor, I think something is wrong with the patient!”
IV or IO access is obtained and the patient is given 1L of IV fluids	The patient will start to wake up. The device returns to normal settings	The patient states: “Oh I don’t feel good. What happened?”

**Table 10:** Ideal scenario flow and anticipated management mistakes.

Ideal scenario flow
Learners enter the room to find a patient moaning and lethargic. His wife is present at the bedside. He has an LVAD, with the controller on his belt. They should obtain a history of recent gastroenteritis from the wife which should make them suspect dehydration. On physical exam, they should auscultate the precordium to appreciate the hum of the device. They should immediately place the patient on the monitor but the automated sphygmomanometer will be unable to read the BP due to the presence of the device. The learners should use the manual sphygmomanometer and vascular Doppler to obtain a mean arterial pressure (MAP). If they do not, the nurse should prompt them to. IV access will be unable to be obtained for this patient until later in the case and the nurse should state that they are trying but the patient is a “tough stick.” The team should attempt to call the LVAD coordinator, and if they do not, the patient’s wife should prompt them to. 10 minutes into the case, the patient should become unresponsive due to a “suction event” causing a PEA arrest. The team should be prompted to re-evaluate if they do not notice, including attempting to obtain a new MAP which should be 0, and evaluate the controller which will show a low flow alarm and high power usage. Standard ACLS should be started including high-quality chest compressions and epinephrine as well as placing the patient on the Zoll. The patient should be intubated and IV access will finally be able to be obtained by the RN. An IV fluid bolus should be administered. If not, the patient will not continue to be in PEA. If given fluids, the patient will wake up. The LVAD coordinator should be called again and the transfer should be arranged to the LVAD center
Anticipated management mistakes
1. Difficulty with evaluating the LVAD: Learners may find it difficult to interact with the controller or not know what the settings mean. They may not know how to use a manual sphygmomanometer and vascular Doppler to obtain a MAP
2. Failure to call the LVAD coordinator: Participants may not know that every LVAD patient has a coordinator on call who can help with the management of any potential emergencies
3. Failure to recognize PEA secondary to the suction event: Learners may not know that the LVAD is preload dependent and dehydration can cause it to stop working, thus necessitating crystalloid resuscitation. They may also not notice that the patient has become unresponsive
